# Anterior capsulotomy and accumbensotomy of obsessive-compulsive disorder with obsessional slowness: a case report

**DOI:** 10.3389/fpsyt.2024.1498046

**Published:** 2024-11-25

**Authors:** Rui Lai, Xiao Pang, Yang Ming, Haiping He, Yu Xiong, Jian You, Ligang Chen, Feilong Gong

**Affiliations:** ^1^ Department of Neurosurgery, Affiliated Hospital of Southwest Medical University, Luzhou, Sichuan, China; ^2^ Neurosurgery Clinical Medical Research Center of Sichuan Province, Luzhou, Sichuan, China; ^3^ Academician (Expert) Workstation of Sichuan Province, Luzhou, Sichuan, China

**Keywords:** anterior capsulotomy, accumbensotomy, obsessional slowness, obsessive-compulsive disorder, stereotactic neurosurgery

## Abstract

Obsessional slowness (OS) is characterized by a syndrome of extreme slowness in doing ordinary, day-to-day activities. Several scholars regarded OS as secondary to obsessive compulsive disorder (OCD). Therefore, it is commonly thought to be the consequence of extensive rituals and has been paid minimal attention in its own right. A combination of behavior therapy and aromatherapy are recommended for treatment of this condition. However, the outcome is often frustrating. Reports of surgical management for OS are limited. Patient concerns:She had symptoms characterized by repeated checking and progressive slowness in self-care behavior. Diagnosis:At the age of 19, the patient had the first presentation. The patient was diagnosed with a case of OCD with obsessional slowness according to the International Classification of Diseases and Related Health Problems (ICD-10).Interventions:Considering the lack of a response to pharmacotherapy and cognitive behavioral therapy (CBT), we treated this case with anterior capsulotomy and accumbensotomy. Outcomes: Moderate somnolence, urticaria, juvenile behavior, mild short-term memory impairment and slight nonsense were noted during the first postoperative days. At 10 months, the patient’s OCD symptoms recovered nearly to her preoperative level. The OS symptom also had an obvious rebound at 10 months. Through comprehensive judgment, we decided to choose accumbensotomy. At 9 months after the accumbensotomy, the OCD symptoms started to rebound. Soon after, the OS symptoms also recurred. At the last timepoint of 30 months, the patient’s OCD and OS symptoms had completely rebounded. This time, the patient and parents refused any treatment. Conclusion: This case suggests that OCD with OS, as a special category, might not be suitable for stereotactic neurosurgery. Furthermore, multiple surgeries in this kind of OCD patient should be considered with as much caution as much as possible.

## 1 Introduction

Obsessional slowness was first described by Rachmanv (1) who documented ten cases of “primary obsessive slowness”. The condition is characterized by prominent debilitating slowness, especially in self-care behavior and extreme meticulousness in doing things. Veale (2) gave rise to the concept of “secondary obsessional slowness”. In previous studies, limited reports focused on obsessional slowness (3, 4).The relatively effective treatments of obsessive slowness are pharmacotherapy and behavioral therapy. Although successful nonoperative management of OCD patients with OS has been reported (5, 6), the evidence for surgical treatment of obsessional slowness in OCD patients is limited. In this report, we present the case of a 22-year-old treatment-refractory OCD female patient with obsessional slowness. The patient had attempted two stereotactic neurosurgeries (anterior capsulotomy and accumbensotomy) in succession, but the outcomes were unsatisfactory. We hope that this case report provide experience to colleagues involved in the management of obsessional slowness.

## 2 Case description

The patient was a 22-year-old unemployed female, with a slowly warm premorbid temperament and pursuing the goal of perfection in everything when she was a child. Her family history was negative for mental disorders. By the age of 16, she was still a junior middle school student, and her performance was always leading the way among her peers. She always made sure every test was absolutely right. Gradually, the patient became extremely meticulous in performing tasks. She would check repetitively every test item, recall repetitively the content of her classes; wonder if she was bringing her textbooks or not and if she had locked or unlocked the door. These features made her do everything slower than others and led to delays in her class. Therefore, she had a gradual academic decline. She started having anxiety and low mood, lost interest in all pleasurable activities and could not find a self-redemption method. Since then, she had a progressive deteriorating course of symptoms characterized by repeated checking (regarding her purse, bag and drawer), repeated fears (something bad would happen to her if she did not perform well at each step in her routine activities) and progressive slowness in self-care behavior (getting up, washing hands, brushing, bathing, walking, and eating). She would divide each act of self-care into numbers of small steps. For each of these steps, she would spend a variable period of time on deciding whether to do it or not and considered the pros and cons of each action. Seeing the patient doing things very slowly, her parents often interrupted her and made decisions for her. These actions of her parents gave her extreme agony and anxiety. She regarded these behaviors as senseless and tried to resist it, but was unable to do so. She considered ending her life twice, but failed. Finally, she stopped going to school and had a rest at home. However, her condition did not improve, and the symptoms worsened.

## 3 Diagnosis

At the age of 19, the patient had the first presentation. The patient could not come in the examination room because she would spend at least 1 hour deciding on whether to step with the left or right foot first. She was taking 1-2 hours getting up, 2-3 hours bathing, 1-2 hours washing her hands and 2-3 hours finishing one meal. The Self-Administered Obsessive Slowness Questionnaire (S-AOSQ) (7) score was 52, and the Yale-Brown Obsessive Compulsive Scale (Y-BOCS) (8), Hamilton Depression Rating Scale (HAM-D) (9), and Hamilton Anxiety Rating Scale (HAM-A) (10) scores, were 37, 17, and 30, respectively, at the time of the initial assessment. The patient was diagnosed with a case of OCD with obsessional slowness according to the International Classification of Diseases and Related Health Problems (ICD-10) (11).

## 4 Treatment

### 4.1 Pharmacotherapy and cognitive behavioral therapy

The patient was started on fluoxetine 20 mg/day for outpatient treatment, and the medication was slowly titrated to 60 mg/day. Initially, the pharmacotherapy had a good effect on remission symptoms. Gradually, tolerance to the drug began to occur. Recurrent symptoms often appeared. At the age of 21 years, the patient was given inpatient treatment and was managed medically with multiple antipsychotics (e.g., paroxetine, clomipramine) in conjunction with cognitive behavioral therapy. However, no significant improvement was achieved at that time. Antipsychotic therapy and cognitive behavioral therapy (CBT) were of limited help in alleviating her symptoms.

### 4.2 Anterior capsulotomy and accumbensotomy

Three months before surgery, due to a lack of response to pharmacotherapy and CBT and concerns about drug side effects, the patient refused further medication, worsening her symptoms The patient and her family started to seek help and counsel for a surgeon. We carefully addressed her treatment options, which included deep brain stimulation, gamma knife or thermocoagulation. Both the patient and her family opted for thermocapsulotomy for several personal reasons. (The patient provided consent for the publication of this paper).

Neuropsychological assessment and psychiatric diagnostics were performed by the same psychiatrist. Preoperative magnetic resonance imaging was performed to rule out an intracranial tumor, hemorrhage, infarction, and infection. Chronicity and treatment refractoriness of OCD for the patient were in concordance with the inclusion criteria for stereotactic neurosurgery in OCD (12). The stereotactic neurosurgical interventions for treatment refractoriness of OCD patients were approved by the institutional review board of the West China Hospital of Sichuan University clinical trials and the biomedical ethics committee. Both the patient and her parents signed informed consent forms.

**Figure 1:**
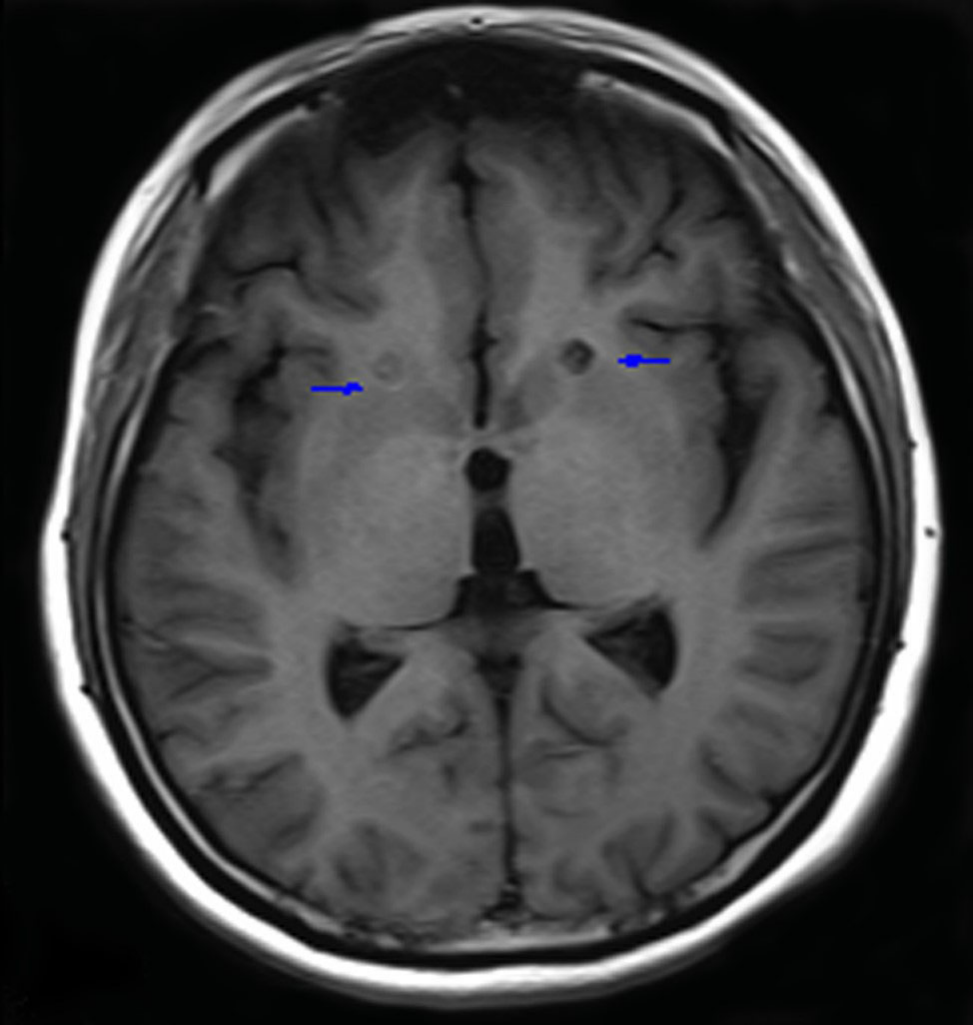
Lesions in the anterior limb of the internal capsule 3 months after surgery. Blue arrows indicate lesions.

Anterior capsulotomy was performed with the patient under local anesthesia and was guided by magnetic resonance imaging (Siemens AG 3.0 T). The lesions were approximately located 14 mm anterior and 18 mm lateral to the anterior commissure and 5 mm below the anterior and posterior commissure plane. Thermocoagulation was performed by heating an electrode connected to a Radionics lesion generator (Elekta) to 75°C for 60 seconds. The same procedure was performed on the other side. The length of the lesions on both sides was 12-14 mm (**Figure 1**).

**Figure 2:**
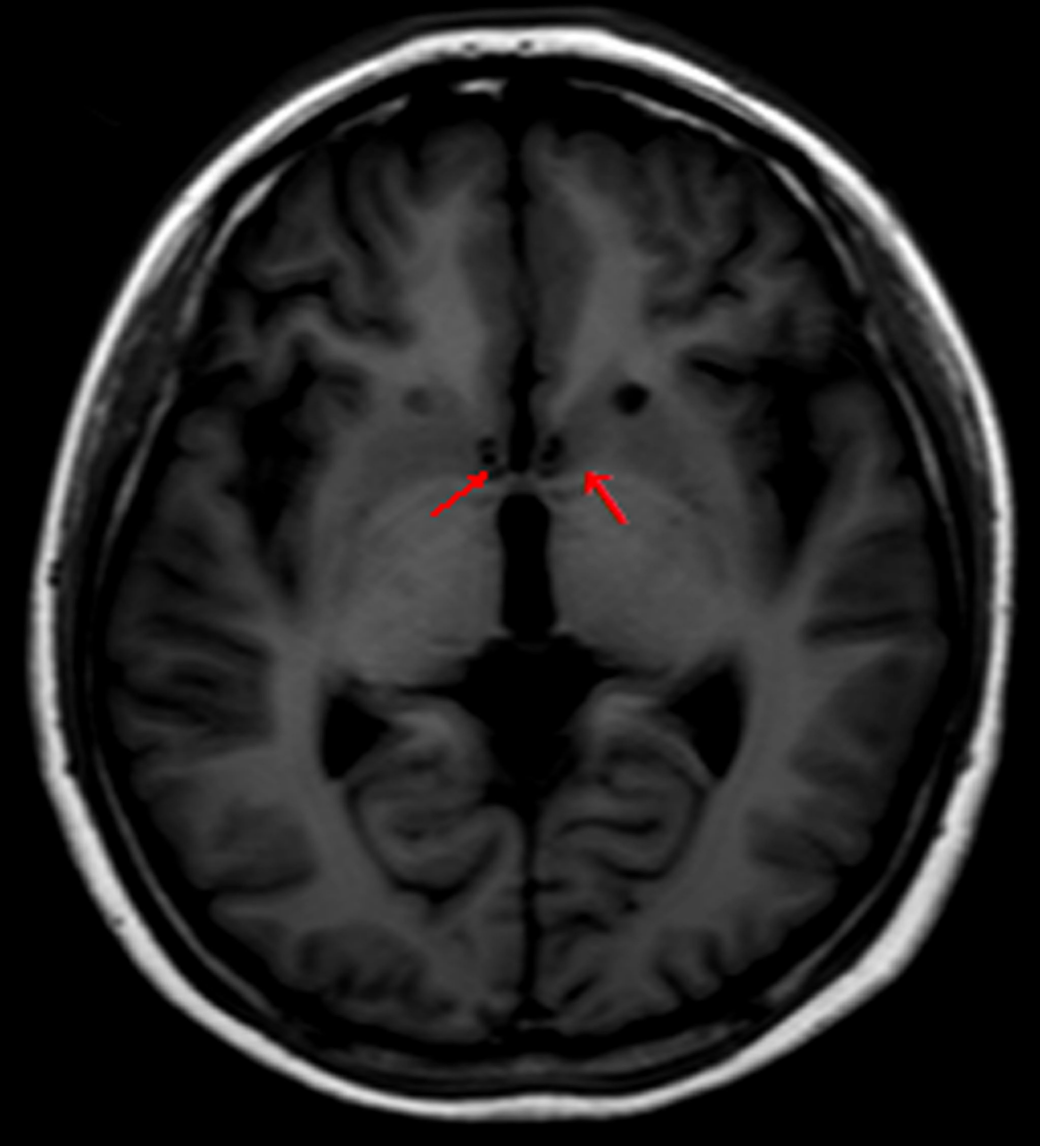
Lesions in the nucleus accumbens 3 months after surgery. Red arrows indicate lesions.

Due to symptom recurrence after the anterior capsulotomy, we performed a second operation 12 months after the first operation. This time, we selected the nucleus accumbens. The lesion targets were located 16 mm anterior and 3 mm lateral to the anterior commissure and 2 mm below the anterior and posterior commissure plane. Other programs had no difference with the capsulotomy (**Figure 2**). Therefore, we gave the patient general anesthesia in the second surgery because of concerns about the patient’s noncooperation.

## 5 Evolution and outcome

### 5.1 Clinical evolution

The clinical symptom evaluations of S-AOSQ, Y-BOCS, HAM-D, HAM-A were performed at baseline, 1 month, 3 months, 6 months, 10 months, 12 months (second operation), 13 months, 15 months, 18 months, 21 months, 24 months, and 30 months (final time point) of follow-up after the first operation by the same psychiatrist in face-to-face or telephone follow-up visits. The patient was considered a responder if a 35% or more reduction in baseline Y-BOCS scores was achieved. The evaluation results of clinical symptoms are shown in **Tables 1**, **2**.

**Table 1:** OCD symptom evaluations at 30-month follow-up.

Test name	First operation(score)					Second operation(score)						
	Baseline	1M	3M	6M	10M	12M	13M	15M	18M	21M	24M	30M
Y-BOCS	37	20	16	24	31	35	34	32	16	33	34	36
HAM-D	17	12	6	10	15	18	13	14	6	12	15	16
HAM-A	30	16	8	12	20	31	28	30	10	24	26	28

BF, before surgery; Y-BOCS, Yale-Brown Obsessive Compulsive Scale; HAM-D, Hamilton Depression Rating Scale; HAM-A, Hamilton Anxiety Rating Scale.

**Table 2:** The self-administered obsession slowness questionnaire at 30-month follow up.

Activity	First operation(score)					Second operation(score)						
	Baseline	1M	3M	6M	10M	12M	13M	15M	18M	21M	24M	30M
Having a bath or shower	3	2	1	1	2	3	3	3	2	3	3	3
Washing hands and face	3	1	1	1	2	3	2	3	1	1	2	3
Care of hair	1	1	0	1	1	1	1	1	0	0	1	1
Brushing teeth	3	2	1	1	2	3	3	3	1	1	3	3
Dressing	3	1	1	1	2	3	3	3	2	2	3	3
Undressing	2	1	0	0	1	1	1	0	0	1	1	1
Using the toilet to urinate	3	3	2	2	2	3	2	3	2	2	2	3
Using the toilet to defaecate	3	3	2	2	3	3	3	3	2	2	3	3
Getting out of bed in the morning	3	2	2	2	2	3	3	3	2	3	3	3
Eating a meal	3	2	2	1	2	3	2	2	1	2	2	3
Cleaning a room in the house	2	1	1	1	1	2	1	1	0	1	2	2
Making a bed	3	2	1	1	2	3	3	3	1	1	3	3
Opening a door by the handle	3	2	1	1	2	3	3	3	2	2	3	3
Switching a light or a tap on or off	3	2	1	1	2	3	3	3	2	2	3	3
Locking or closing a door or window	3	2	2	1	2	3	2	3	2	2	2	3
Writing a letter to a friend	1	1	0	0	1	1	1	1	0	0	1	1
Putting a letter in the post-box	1	1	0	0	1	1	1	1	0	0	1	1
Walking from one room to another	3	1	0	0	2	3	3	3	1	2	3	3
Walking 100 yards down the street	3	1	0	0	2	3	3	3	1	2	3	3
Crossing the road	3	1	0	0	2	3	3	3	1	2	3	3
Total scores	52	32	18	18	36	51	46	48	23	30	47	51

Instructions: It lists 20 common items of behavior. The patient is asked to rate the speed of her own performance of each itemin comparison with an average person. The scoring method is shown on the questionnaire.0 score: I have no problem with this activity-it takes me about the same time as the average person. 1 score: This activity takes me about twice as long as most people. 2 score:This activity takes me about three times as lone as most people. 3 score. This, activity takes me more than three times as long as most people. The maximum score is 60, corresponding to the maximum severity of subjective slowness. A score of 30 was chosen as the criterion score for the purpose of the study.

### 5.2 Outcome

As shown in **Table 3**, after the first operation, the S-AOSQ, Y-BOCS, HAM-D, and HAM-A scores respectively decreased by 38.46%,45.95%, 29.41% and 46.67% at one month, OS symptoms improved significantly. Moderate somnolence, urticaria, juvenile behavior, mild short-term memory impairment and slight nonsense were noted during the first postoperative days. Most of these complications disappeared in the first 2 postoperative weeks. A stable personality change was present at one month after the capsulotomy. The personality change showed features such as the following: apathy, being indifferent to the things that were important for her before and being able to reconcile herself to any situation; laziness, such as not paying attention to personal hygiene and her own care; laughing quietly, often laughing at common things or laughing by herself with no stimulation. Moreover, this personality change was proven to be irreversible with passing time. The postoperative Y-BOCS, HAM-D, and HAM-A scores respectively decreased by 65.38%,56.76%,64.71% and 73.33% at 3 months. At the 3-month timepoint, the OS symptoms were in complete remission. The patient could move on her own, getting up in 50 minutes, bathing in 1 hour, brushing teeth for 30 minutes, washing hands one or two times, and finishing one meal in 50 minutes. At the 6 month, the S-AOSQ, Y-BOCS, HAM-D, and HAM-A scores respectively decreased by 67.31%,35.14%,41.18% and 60.00%, the OCD symptoms rebounded. However, there was no obvious recurrence of the OS symptom. Unfortunately, a new problem of sexual disinhibition presented. The patient had a strong demand for sex. She always wanted to have a contact with a member of the opposite sex and fall in love with them. This symptom became serious at 6 months. She became continuously acquainted with a member of the opposite sex in her network and was dating him. Without telling her families where she was going, she usually disappeared for a few days and was cohabitating with her boyfriend. The patient did not answer phone calls and was lying to her parents. Her character became impulsive and irritable. At 10 months, the S-AOSQ, Y-BOCS, HAM-D and HAM-A scores respectively decreased by 30.77%,16.22%,11.76% and 33.33%,the patient’s OCD symptoms recovered nearly to her preoperative level. The OS symptom also had an obvious rebound at 10 months. The patient began to have difficulty in moving forward, and the self-care time was prolonged.

**Table 3:** The difference comparison between Y-BOCS,HAM-D,HAM-A and S-AOSQ scores at 30-month follow up.

Test name	First operation(score)					Second operation(score)						
	Baseline	1M	3M	6M	10M	12M	13M	15M	18M	21M	24M	30M
S-AOSQ	52	38.46%	65.38%	67.31%	30.77%	1.92%	11.54%	7.69%	55.77%	42.31%	9.62%	1.92%
Y-BOCS	37	45.95%	56.76%	35.14%	16.22%	5.41%	8.11%	13.51%	56.76%	10.81%	8.11%	2.70%
HAM-D	17	29.41%	64.71%	41,18%	11.76%	-5.88%	23.53%	17.65%	64.71%	29.41%	11.76%	5.88%
HAM-A	30	46.67%	73.33%	60.00%	33.33%	-3.33%	6.67%	0.00%	66.67%	20.00%	13.33%	6.67%

At 12 months, the S-AOSQ and Y-BOCS scores respectively decreased by 1.92%,5.41%.However, the HAM-D and HAM-A scores respectively increased 5.88% and 3.33%. Through comprehensive judgment, we determined that the patient had a relapse. The patient and her parents refused to take any medicine or have behavior therapy. They finally preferred a re-operation. We carefully addressed her operation program and considered her new symptom of sexual disinhibition. Accumbensotomy was performed 12 months after the anterior capsulotomy. Transient complications of the first surgery were not observed during the second postoperative days, except mild somnolence. The sexual disinhibition disappeared completely by the first week after surgery. At 13 months, the S-AOSQ, Y-BOCS, HAM-D and HAM-A scores respectively decreased by 11.54%,8.11%,23.53% and 6.67% after Accumbensotomy compared with before the first surgery. At 15 months, the S-AOSQ, Y-BOCS and HAM-D scores respectively decreased by 7.69%,13.51% and 17.65%.However, the HAM-A scores reverted to the initial pre-operative level. Neither OCD nor OS symptoms were obviously alleviated until 6 months after accumbensotomy. At the 18 month, the S-AOSQ, Y-BOCS, HAM-D, and HAM-A scores respectively decreased by 55.77%,56.76%,64.71% and 66.67%. The OCD symptoms were reported to be in obvious remission. This situation did not continue for much time. At the 21 month, the S-AOSQ, Y-BOCS, HAM-D, and HAM-A scores respectively decreased by 42.31%,10.81%,29.41% and 20.00%, the OCD symptoms started to rebound. Soon after, the OS symptoms also recurred. At the 21 month, the S-AOSQ, Y-BOCS, HAM-D, and HAM-A scores respectively decreased by 9.62%,8.11%,11.76 and 13.33%. At the last timepoint of 30 months (18 months after accumbensotomy), the patient’s OCD and OS symptoms had completely rebounded, with Y-BOCS, HAM-D, and HAM-A scores decreased by 1.92%,2.70%,5.88% and 6.67%,respectively. As with her preoperative status, the patient could not do anything without someone’s help. This time, the patient and parents refused any treatment.

## 6 Discussion

Several authors (2, 13) suggest that there is a subset of other obsessions in obsessional slowness, hence giving rise to the concept of “primary” and “secondary” obsessional slowness, and they stated that most of the cases belonging to this syndrome suffer from OCD with secondary slowness. Pharmacotherapy or/and behavioral therapy was/were the main treatment methods in previous reports. Rachman (1) has described behavioural therapy for OS, which has been replicated by case studies (2, 25, 26). In these cases, interventions sped up some specific slow symptoms; Nevertheless, the effects were constrained, and the majority of participants exhibited signs of relapse shortly after the therapy was tapered. Singh G (5) described a case of a 21-year-old male patient who presented with debilitating slowness and responded to a combination of behavior therapy (thought habituation and exposure) and pharmacotherapy (fluoxetine and thyroxine). Mittal A K (6) reported a case of early onset severe OCD with obsessive slowness that showed good response to combined pharmacotherapy and behavioral therapy in the form of prompting, pacing, and shaping. This suggests that a combination of pharmacotherapy and behavioral therapy may be more effective.

Recent research raises questions about the classification of obsessional slowness (OS) as a ‘primary’ disorder, suggesting it may represent a more severe manifestation of obsessive-compulsive disorder (OCD). This perspective could significantly impact treatment decisions for these patients, advocating for the consideration of established OCD therapies (3, 4). Beginning in the 1980s, researchers embarked on investigating the biological underpinnings of OCD, leading to the subsequent discovery of the cortico-striato-thalamo-cortical (CSTC) loops. This intricate network comprises distinct basal ganglio-thalamocortical circuits that emanate from specific regions within the prefrontal cortex and extend to corresponding targets in the striatum and thalamus (14). The CSTC model postulates that excessive activity within these frontal-subcortical circuits forms the biological foundation for OCD symptoms (15, 16). Notably, anterior limb of the internal capsule(ALIC) plays a significant role in facilitating bidirectional connections between the prefrontal cortex and both the striatum and thalamus within this circuitry. Consequently, employing ALIC as a target in psychosurgery is a reasonable approach. Anterior capsulotomy, aimed at the anterior limb of the internal capsule, is believed to interfere with communication between the orbitofron-tal cortex (OFC), dorsal anterior cin-gulate cortex (dACC), ventral striatum, and thalamus, its ablation yielded treatment efficacy of 40% to 80% (17, 18). A recent systematic review of observational studies involving 193 participants and 10 studies has shown that at a 12-month follow-up, the mean reduction in the Y-BOCS score was 55% for capsulotomy. At the last follow-up, the mean reduction in Y-BOCS score was 57% for capsulotomy (19). Some scholars (2, 13) found that that there was no evidence either on brain pathology or neuropsychology, and no multivariate analysis has been able to delineate a subgroup of slowness symptoms within the syndrome of OCD. Therefore, capsulotomy might also improve obsessive slowness symptoms at the same time. However, in rare case reports, there has been surgical therapy for OCD with obsessive slowness. Typical capsulotomy has been used mostly for intractable OCD in the past 5 decades. This hypothesis has been proven to be partly correct. The patient did present a significant improvement in OCD symptoms as well as remission of obsessive slowness for a few months after capsulotomy.

Obvious sexual disinhibition appeared 6 months after capsulotomy. Thereafter, OCD symptoms began to rebound, but the OS symptom did not. We assumed that the patient’s obsessive slowness might be secondary to severe OCD symptoms. As expected, the OCD symptoms had a complete relapse at 10 months, and the obsessive slowness was also aggravated. According to a previous study, Rück C et al. (17) reported that one man who underwent thermocapsulotomy was severely sexually disinhibited immediately after surgery and was subsequently convicted of rape 5 months postoperatively. Sexual disinhibition as a manifestation of personality change is a major complication of capsulotomy, which might lead to grave consequences.

Through comprehensive analysis, we came to the following possible conclusions about the first relapse. First, the patient did not completely cooperate during the whole operation under local anesthesia, which led to incomplete ablation in the right anterior limb of the internal capsule. Lippitz et al. (17) found that capsulotomy in the right hemisphere was decisive for a favorable therapeutic outcome. Second, the broken nerve fibers that pass through the anterior limb of the internal capsule might have incomplete repair; therefore, OCD symptoms recurred. Third, as a special type of treatment-refractory obsessive–compulsive disorder with obsessive slowness, there could be other unknown loops besides the classic cortico-striato-thalamo-cortical (CSTC) pathway, in which the capsulotomy could not intervene. N. HYMAS believes that patients with obsessional slowness may have a dysfunction in the frontal-basal-ganglia loop system (7). Therefore,the patients who experienced anterior capsulotomy exhibited a persistent and recurring obsessional slowness symptoms even after 10 months of remission.

Considering the above three suppositions and new symptom of sexual disinhibition, we selected the nucleus accumbens (NAC) as the target for the second surgery. The nucleus accumbens has been proven to have a reaction to the brain’s happiness centers, such as food, sex, and drugs. The underlying pathogenetic mechanism of OCD is caused by a failure of inhibition of the ventral striatum (20). The nucleus accumbens is part of the ventral striatum. Therefore, an accumbensotomy might affect the ventral striatum through the projectional fibers from the ventral striatum and result in significantly improved OCD symptoms. In the past decade, there were constant reports about the deep brain stimulation of the nucleus accumbens for treatment-refractory obsessive-compulsive disorder. In these reports, this procedure was proven to have significant improvements in reducing OCD symptoms and had less adverse reactions than those of capsulotomy (21, 22). Specifically, the primary advantage of deep brain stimulation (DBS) over ablative neurosurgery lies in its capacity to facilitate bilateral procedures within the motor regions of the basal ganglia and thalamus while minimizing the risk of adverse effects on speech, swallowing, cognition, and balance. However, a comprehensive literature review comprising 20 studies indicated that patients undergoing Anterior capsulotomy exhibited a 9% higher likelihood of achieving remission compared to those receiving DBS, while no significant disparities were observed in terms of complication rates (23).

Typical side-effects were not observed after accumbensotomy except mild somnolence. This result was consistent with several previous reports (22, 24). Nevertheless, the OCD and OS symptoms did show remission. Fortunately, the severe sexual disinhibition disappeared completely by the first week after the second surgery, as expected. Significant improvements in symptoms occurred at 6 months after accumbensotomy. However, the symptoms relapsed only 3 months later.

## 7 Conclusion

**Figure 3:**
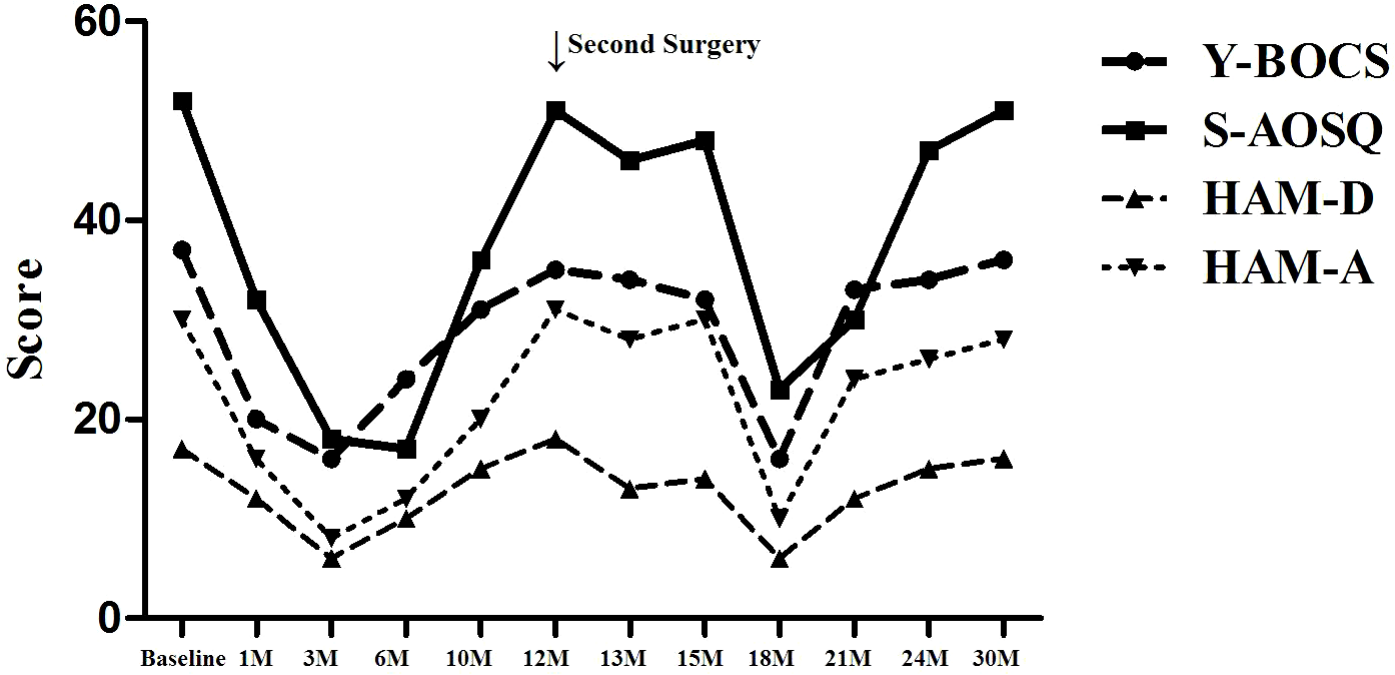
The comparison between Y-BOCS,HAM-D,HAM-A and S-AOSQ scores at 30-month follow up.

We report a case of a 22-year-old female OCD patient with OS. After anterior capsulotomy and accumbensotomy, her obsessive-compulsive symptoms and obsessional slowness symptoms both presented a transient remission, but they relapsed (**Figure 3**). This outcome is a significant case for us in terms of learning. There are several questions that we should contemplate. First, OCD patients with OS might not be suitable for thermocoagulation or radiosurgery. For this special patient, a relatively reversible surgery method, such as DBS, might be a good choice. DBS can significantly relieve OCD symptoms, the non-ablative, reversible, and more tolerant nature of DBS makes it a more acceptable treatment option. Our department is attempting this procedure. Second, there might be other loops besides the classic cortico-striato-thalamo-cortical (CSTC) pathway for this special type of OCD, such as frontal-basal-ganglia loop system. Further research is required on this topic. Third, not all the treatment-refractory obsessive–compulsive disorder handle surgical interventions, especially when accompanied by compulsive slowness. According to our limited clinical experience, OCD patients who mainly experienced compulsive behavior would have a much better surgery remission than that of patients with compulsive thoughts, for the latter, pharmacotherapy combined with cognitive behavioral therapy may be considered. For some special patients, such as OCD with mental symptoms or OCD with and other symptoms, surgery requires careful consideration. Priority should be given to DBS therapy targeting the ventral sac/ventral striatum and nucleus accumbens for OCD patients. The pathological mechanism of OS needs further study.

## Data Availability

The original contributions presented in the study are included in the article/supplementary material. Further inquiries can be directed to the corresponding authors.
